# Key Components of Inflammasome and Pyroptosis Pathways Are Deficient in Canines and Felines, Possibly Affecting Their Response to SARS-CoV-2 Infection

**DOI:** 10.3389/fimmu.2020.592622

**Published:** 2021-01-28

**Authors:** Haoran Cui, Leiliang Zhang

**Affiliations:** ^1^ Institute of Basic Medicine, The First Affiliated Hospital of Shandong First Medical University, Jinan, China; ^2^ Science and Technology Innovation Center, Shandong First Medical University & Shandong Academy of Medical Sciences, Jinan, China

**Keywords:** Severe Acute Respiratory Syndrome Corona Virus 2, canines, inflammasome, felines, pyroptosis

## Abstract

SARS-CoV-2 causes the ongoing COVID-19 pandemic. Natural SARS-COV-2 infection has been detected in dogs, cats and tigers. However, the symptoms in canines and felines were mild. The underlying mechanisms are unknown. Excessive activation of inflammasome pathways can trigger cytokine storm and severe damage to host. In current study, we performed a comparative genomics study of key components of inflammasome and pyroptosis pathways in dogs, cats and tigers. Cats and tigers do not have AIM2 and NLRP1. Dogs do not contain AIM2, and encode a short form of NLRC4. The activation sites in GSDMB were absent in dogs, cats and tigers, while GSDME activation sites in cats and tigers were abolished. We propose that deficiencies of inflammasome and pyroptosis pathways might provide an evolutionary advantage against SARS-CoV-2 by reducing cytokine storm-induced host damage. Our findings will shed important lights on the mild symptoms in canines and felines infected with SARS-CoV-2.

## Introduction

Corona Virus Disease 2019 (COVID-19) caused by Severe Acute Respiratory Syndrome Corona Virus 2 (SARS-CoV-2) is an ongoing global pandemic, with over 15 million confirmed cases as of July 25^th^, 2020. Rhinolophus sinicus (Chinese rufous horseshoe bat) is well accepted as the original host of SARS-CoV-2 (1). Pangolin is proposed as a potential intermediate host for SARS-CoV-2 (2, 3). Felines (cats and tigers) and canines (dogs) have been reported to be infected by SARS-CoV-2 (4–8). Given the zoonotic origin of the virus, it is of great public health interest to investigate the pathology of the potential animal reservoirs. Notably, the clinical respiratory sign in canines and felines are milder compared to human counterpart, however the underlying mechanisms of which are currently unknown.

Inflammasomes consist of sensor molecules, the adaptor protein ASC, and the downstream effector caspases. Inflammasome sensor molecules are classified into PYHIN (AIM2 and IFI16) and NLR (NLRP1, NLRP3, NLRP6, NLRP9, NLRP12 and NLRC4) (9). Activation of sensors results in caspase activation, which cleaves the precursors of the inflammatory cytokines Interleukin (IL)-1β and IL-18 into their active forms. Gasdermins (GSDMs) belong to a protein superfamily that consists of GSDMA, GSDMB, GSDMC, GSDMD, GSDME and DFNB59 in humans (10). GSDMs possess pore-forming activity that mediate a regulated lytic cell death mode termed pyroptosis (10). Pyroptosis controls inflammasome-dependent cytokine secretion and contributes to antimicrobial defense and inflammasome-mediated autoinflammatory diseases.

Several clinical studies showed that increased inflammasome activity leads to immune dysregulation and ultimately severe disease for COVID-19 patients (11–13). A recent study directly demonstrates that the NLRP3 inflammasome is activated in response to SARS-CoV-2 infection (14). Inflammasomes and pyroptosis have been proposed as therapeutic targets for COVID-19 (15). How inflammasome and pyroptosis in canines and felines differ from human is worth studying.

To cope with viral infections, mammals have evolved elegant tolerance strategies to reduce excessive inflammatory damage while sustaining the virus replication. RNA viruses activate sensors for intracellular RNA, such as IFIH1/MDA5, ZBP1, and DDX58/RIG-I. Pangolins have lost IFIH1/MDA5 or ZBP1 to dampen innate immunity (16). cGAS senses DNA and catalyzes the production of 2′3′-cGAMP, the ligand of STING. STING-dependent IFN activation is suppressed in bats due to the replacement of the functionally important serine residue S358 (17). cGAS and STING have been inactivated by mutations in the Malayan pangolin, Chinese pangolins, and tree pangolins (18). NLRP3 inflammasome plays a critical role in the immune response to viruses. Multiple mechanisms attribute to dampened NLRP3-mediated inflammation in bats (19). Members of the PYHIN family are DNA sensors capable of recognizing DNA viruses and damaged own DNA. The absence of the PYHIN family in bats may avoid excessive inflammation against damaged self-DNA generated during RNA viral infection (20).

The purpose of the present study was to investigate the key components of inflammasome and pyroptosis pathways in canines and felines and compared them with that in human. We found natural deletion or functional loss of critical inflammasome and pyroptosis components in cat, tiger and dog, implicating deficiency of innate defense and partially explaining the mild symptoms of SARS-CoV-2 infection in canines and felines.

## Material and Methods

### Cell Culture

Madin-Darby canine kidney (MDCK) cell line were preserved by our laboratory and cultured in DMEM with 10% FBS. Cells were grown at 37°C with 5% CO_2_/95% air atmosphere and were revived every 3 to 4 months.

### RNA and DNA Extraction

MDCK cell DNA were extracted using mammalian genomic DNA extraction kit (Beyotime) according to the manufacturer’s protocol. The total RNA was isolated from the MDCK cells using Invitrogen TRIzol Reagent. RNA concentrations and A_260_/A_280_ ratios were determined using a NanoDrop Spectrometer. cDNA fragments were synthesized from total RNA using the PrimeScript^™^ II 1st Strand cDNA Synthesis Kit (Takara).

### PCR Amplification

Nested PCR was utilized to improve the specificity of PCR product. The primers designed based on target gene sequences were listed in **Tables 1** and **2**. PCR were performed with a Veriti thermocycler (ABI) using 2×EasyTaq PCR SuperMix (Transgen).

**Table 1:** The nested primers used in dog cDNA amplification.

Nested primers for dog cDNA amplification	
inner Dog NLRC4 F	CTTACAGAAAATGGCTTTCA
inner Dog NLRC4 R	CTAAAGCAAAACTATATGATACCTC
outer Dog NLRC4 F	GGGAAAGTCACTTACAGAAA
outer Dog NLRC4 R	CTAAAGCAAAACTATATGATACCTC
inner Dog IFI16 F	ATGGAGGGTGAGTACAAGAA
inner Dog IFI16 R	TCATGGTGAGGTTTCCATAT
outer Dog IFI16 F	ACACCTGGAGATGGAGG
outer Dog IFI16 R	CTTAGAAGGACATCATGGTGAG

F means forward primer, while R means reverse primer.

**Table 2:** The nested primers used in dog DNA amplification.

Nested primers for dog DNA amplification	
inner Dog GSDMB F	TTAAGCATCTGACTCTTGGT
inner Dog GSDMB R	TCATTGTCTCCTGCTAACC
Outer Dog GSDMB F	CTCAGTTAGTTAAGCATCT
Outer Dog GSDMB R	CATCCATCATCATTGTCT

F means forward primer, while R means reverse primer.

### Sequence Analysis of Inflammasome Pathways in Cat, Tiger, Dog, and Human

Genome sequences of human, dog, cat and tiger were downloaded from NCBI. The NCBI accession numbers were as follow: GCF_000004105.39 for *Homo sapiens* (human), GCF_003251725.1 for *Canis lupus familiaris* (dog), GCF_000181335.3 for *Felis catus* (cat), GCF_000464555.1 for *Panther tigris* (tiger). Local BLAST was conducted by Bioedit. Chromosomal locations of each gene were obtained by genome annotation file provided by NCBI. Sequence alignment were conducted by R package DECIPHER (21) and visualized in MEGA X (22).

### Conserved Domain Analysis and Structure Simulation of Dog NLRC4

Conserved domains were identified by NCBI domain search (23). The 3D structure of dog NLRC4 was obtained through homolog modeling using swiss-model (https://swissmodel.expasy.org/) (24). The template was downloaded from PDB database with PDB ID 4KXF (25). The superimposed image was generated by chimera software (Downloaded from http://www.rbvi.ucsf.edu/chimera) (26).

## Results

### PYHIN Family in Dog, Cat, and Tiger Genomes

**Figure 1:**
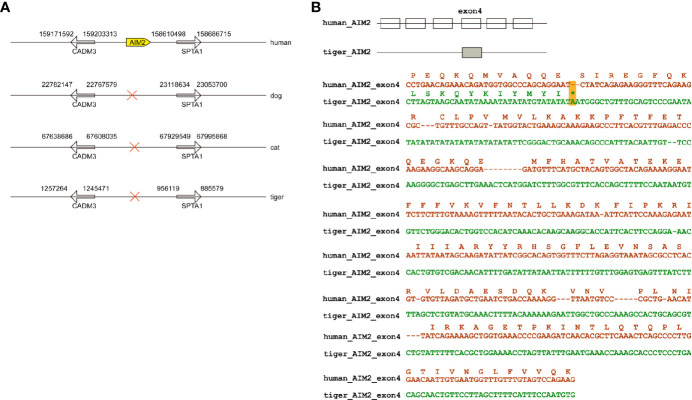
Natural deletion of *AIM2* in tiger genome. **(A)** *AIM2* genes are not present in dogs, cats, and tigers. **(B)** Exons are represented by boxes. Nucleotide sequences of tigers and humans were aligned. In-frame stop codon is highlighted by yellow shading.

Members of *PYHIN* gene family were a cluster of genes in chromosome located between *SPTA1* and *CDMA3* genes (**Figure 1A**) (27). PYHIN proteins, especially AIM2 and IFI16 are viral infection sensors and activate innate immune response in human cells (28). Previously studies have demonstrated that no AIM2 existed in dogs and cats (18, 29). Similar to dogs and cats, tigers have been reported to be infected by SARS-CoV-2 with mild clinical signs (8). Through genome screening, we found that *AIM2* were absent in tiger genome (**Figure 1A**). Local BLAST against tiger genome using human *AIM2* sequence was conducted and only exon 4 was found in tiger genome. Sequence alignment between human and tiger *AIM2* exon 4 showed that several frame-shift mutations, including a stop codon appeared in tiger *AIM2* (**Figure 1B**).

**Figure 2:**
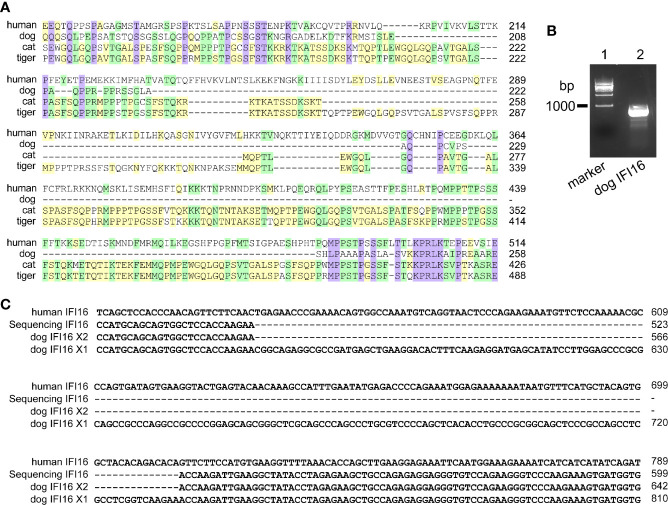
IFI16 from dogs, cats, and tigers are shorter than human IFI16. **(A)** Partial sequence alignment of IFI16 from humans, dogs, cats and tigers. **(B)** PCR amplification of dog IFI16 from Madin-Darby canine kidney (MDCK) cell cDNA. Lane 1, marker of 1,000 bp ladder. Lane 2, band of dog IFI16. **(C)** Partial sequence alignment of PCR product sequence and dog IFI16 isoforms retrieved from annotated genome.

Human IFI16 contains HIN-200A domain (amino acid residues 171-287) and HIN-200B domain and (amino acid residues 499-729) to recognize nucleic acids. In dogs, cats and tigers, IFI16 proteins are truncated in HIN-200A domain compared with human IFI16 (**Figure 2A** and **Figure S1**). To further verify the deficiencies of IFI16, coding sequence of dog IFI16 were amplified using MDCK cell RNA as template, and aligned with annotated dog genome (**Figure 2B**). Sequencing alignment showed that PCR product were consistent with interferon-activable protein 203 isoform X2 in dog genome, as shown in **Figure 2C** and **Figure S1**.

Taken together, PYHIN family members AIM2 and IFI16 from dogs, cats and tigers were deficient.

### NLR Family in Dog, Cat, and Tiger Genomes

**Figure 3:**
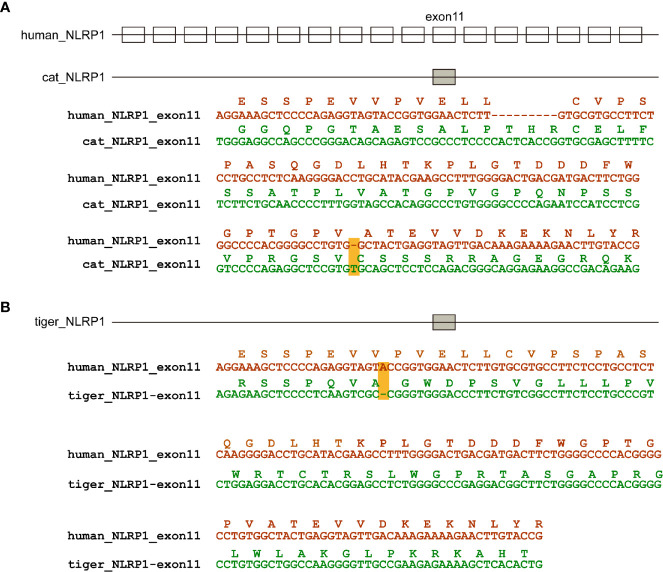
NLRP1 were absent in cat and dog genomes. **(A)** Natural deletion of NLRP1 in cat genomes. **(B)** Natural deletion of NLRP1 in tiger genomes. Exons are represented by boxes. Nucleotide sequences of cat, tiger and human were aligned. Amino acids encoded by exon 11 are shown as boxes. Frame-shift mutations are highlighted by yellow shading.

According to genome annotation, *NLRP3, NLRP6, NLRP9*, and *NLRP12* sequences were present in dog, cat and tiger genomes (**Figures S2–S9**). Interestingly, *NLRP1* sequences were existed in dog, but absent in cat and tiger genomes (**Figures S10** and **S11**). Genome data viewer showed that compared with human, genomes of cat and tigers lack *NLRP1* genes in the corresponding region (**Figures S12** and **S13**). Local BLAST against tiger genome using human *NLRP1* sequence showed that exon 11 existed in cat and tiger genome, with several frame shift mutations in their sequences (**Figure 3**).

**Figure 4:**
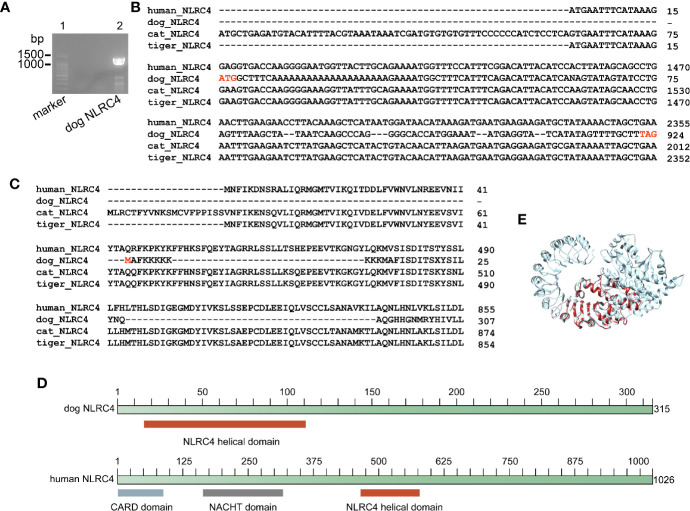
Dog NLRC4 is a truncated protein without CARD domain and NACHT domain. **(A)** PCR amplification of dog NLRC4 from MDCK cell cDNA. Lane 1, marker of 100bp ladder. Lane 2, band of dog NLRC4. **(B)** Alignment of NLRC4 nucleotide sequences from humans, dogs, cats and tigers. **(C)** Alignment of NLRC4 protein sequences from humans, dogs, cats and tigers. **(D)** Diagram of NLRC4 domains from humans and dogs. **(E)** Super impose of NLRC4 tertiary structure from humans and dogs. Human NLRC4 and dog NLRC4 are in orange red and grey, respectively.

Genome analysis demonstrated that dog, cat and tiger contain *NLRC4* gene, as well as human. Sequence alignment showed that NLRC4 from cats and tigers contain similar NLRC4 as human (**Figures S14** and **S15**). Consistent with previous study, a shorter *NLRC4* gene was found in dog through genome screening (30). To verify the results, nested PCR primers were designed to amplify NLRC4 from dog cDNA. Sequencing on PCR products showed that dog *NLRC4* gene were existed, shorter than human *NLRC4* (**Figures 4A, B**).

Conserved domain searching revealed that dog NLRC4 protein lacked CARD domain and NACHT domain compared with human NLRC4 (**Figures 4C**, **D**). Tertiary structure of NLRC4 protein was simulated using human NLRC4 crystal structure as a template (PDB ID 4kxf). The superimposed image showed that dog NLRC4 only contained NLRC4 helical domain and part of LRR-RI domain (**Figure 4E**). The CARD domain of NLRC4 could activate caspase-1 (CASP1) by initiating pro-CASP1 oligomerization (31). The NACHT domain of NLRC4 could hydrolyze ATP to activate NLR-inflammasome (31). Thus, dog NLRC4 protein will be deficient in activate inflammasome. Taken together, cats and tigers do not encode *NLRP1* and dog contains a truncated form of NLRC4, indicating there is deficiency of NLR family in canines and felines.

### ASC, CASP1, and Gasdermin Family in Dog, Cat, and Tiger Genomes

ASC, also called PYCARD, is involved in virus infection. Sequence alignment of ASC protein and mRNA sequences showed that the genes were conserved in dogs, cats and tigers (**Figures S16** and **S17**). For downstream effector CASP1, alignment among humans, dogs, cats and tigers were performed. Tigers contained a longer isoform of CASP1 compared with other species (**Figures S18** and **S19**).

Gasdermin family contained five members, GSDMA, GSDMB, GSDMC, GSDMD and GSDME. According to genome annotation, *GSDMA, GSDMC*, and *GSDMD* sequences were present in dog, cat and tiger genomes (**Figures S20–S25**). Although whether GSDMA and GSDMC were involved in pyroptosis is not clear, GSDMD from dogs, cats and tigers maintained intact cleavage sites for caspases.

**Figure 5:**
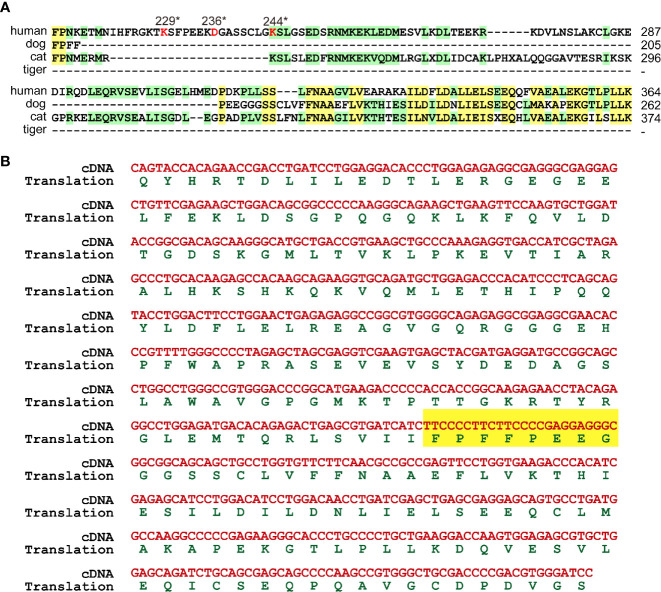
GSDMB were deficient in cleavage sites. **(A)** GSDMB from dogs, cats and tigers lose the cleavage sites of CASP1 and granzyme **(A)** Protein sequences of GSDMB from humans, dogs, and tigers were aligned. The key cleavage sits are marked in red. **(B)** Sequence of dog GSDMB PCR amplification production and the deduced protein sequence were shown.

Human GSDMB was cleaved by CASP1 and granzyme A at D236 (32) and K229/K244 (33) respectively. Sequence alignment showed that cat GSDMB only preserved K256 corresponding to K244 of human GDSMB (**Figure 5A**, **Figure S26**). Dog and tiger GSDMB lack all three potential cleavage residues (**Figure 5A**). MDCK cell DNA was used as template to confirm the deficiencies in GSDMB. Sequence alignment between amplified fragment and dog genome revealed that dog GSDMB lacks the cleavage sites for CASP1 and granzyme A (**Figure 5B**).

**Figure 6:**
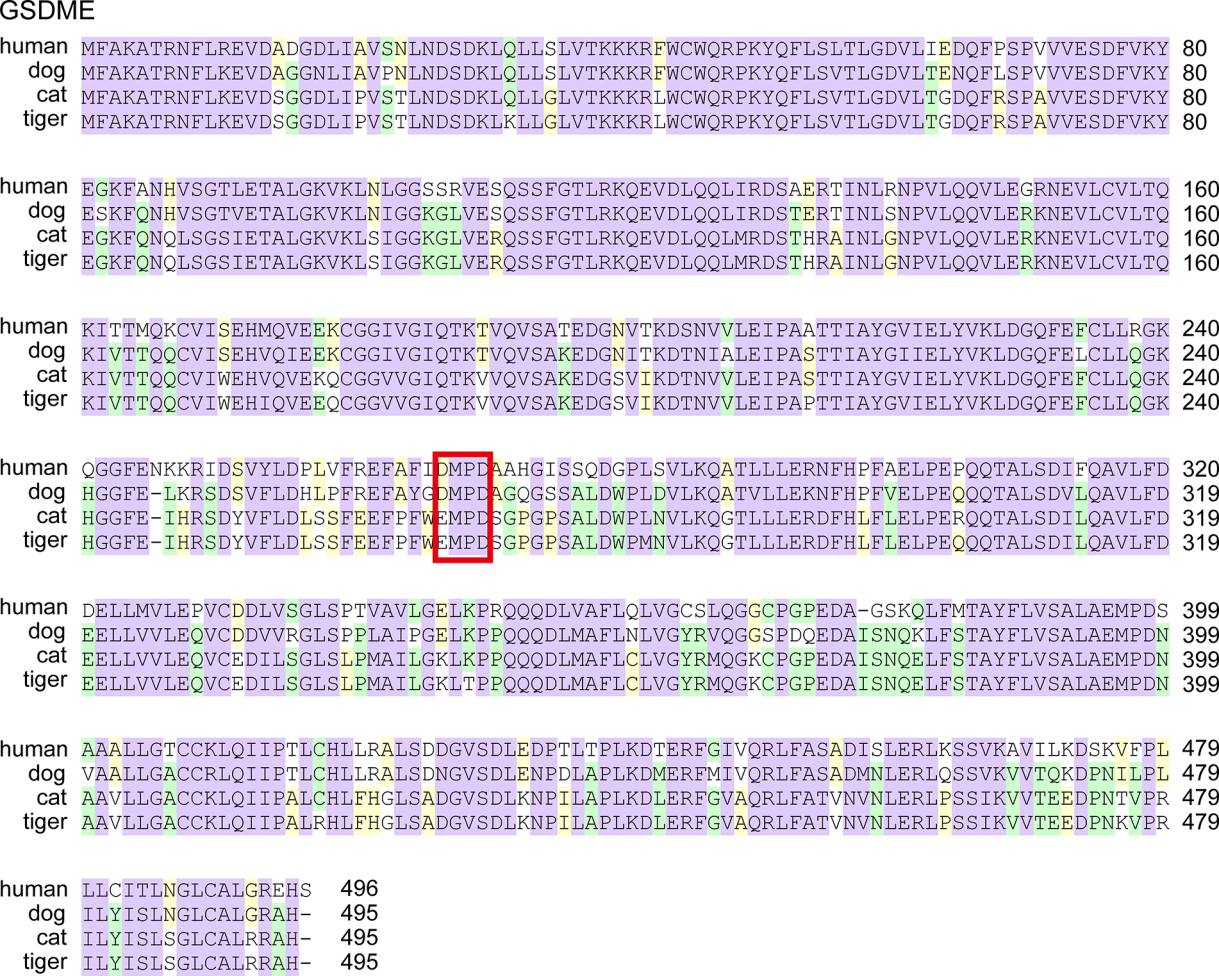
GSDME from cats and tigers lose the cleavage sites of caspase-3 and granzyme B Protein sequences of GSDME from humans, dogs, and tigers were aligned. The key cleavage sits are boxed in red.

Cleavage of GSDME protein by caspase-3 required _267_DMPD_270_ or _267_DMLD_270_ motif (34, 35). Granzyme B also cleaved GSDME at the same site as caspase-3 (36). As shown in **Figure 6**, dog GSDME contained DMPD motif, while in cat and tiger, the amino acids in corresponding sites turned into EMPD. Taken together, some gasdermin family members GSDMB and GSDME from dog, cat and tiger were deficient.

## Discussion

Canines and felines have attracted attention recently because they could be infected by SARS-CoV-2 (4–8). ACE2 proteins from dogs, cats and tigers were predicted to interact with SARS-CoV-2 S protein and supported SARS-CoV-2 entry (37, 38). However, the phenomenon of mild symptoms in these mammals required an explanation. We proposed that few or no clinical disease in SARS-CoV-2-infected canines and felines was likely due to an intricate interplay between the host immune system response and virus infection. Through comparative genome analysis, we discover that the loss of some elements of inflammasome and pyroptosis pathways could be partially responsible for that intricate balance.

Viruses could trigger overreactions of the immune system which cause more harm to the hosts than the viruses itself. Excessive activation of inflammasome pathways can trigger uncontrolled secretion of pro-inflammatory cytokines, leading to cytokine storm and severe damage to host. As inflammasome pathways have recently been recognized to be a central player in viral infection (9), the differences in these pathways between humans and canines/felines may play a role in differential outcoming of COVID-19 infection. Our data suggested that several canines/felines proteins involved in inflammasome pathways are differ from humans. The deficiency of proteins in dogs, cats and tigers was summarized in **Table 3**. Dog NLRC4 protein is truncated, whereas cat and tiger do not have NLRP1. Consistent with previous studies (18, 29), cat and dog do not encode AIM2. Moreover, tiger does not contain AIM2 either. Taken together, canines and felines are deficient in inflammasome sensors, which could tolerate the viral infection.

**Table 3:** The deficiencies of key components of inflammasome pathway in dogs, cats, and tigers.

Summary of the deficiencies of inflammasome components				
Component		Dog	Cat	Tiger
ASC		–	–	–
Caspase1		–	–	–
NLR	NLRP1	–	Absent	Absent
	NLRP3	–	–	–
	NLRP6	–	–	–
	NLRP9	–	–	–
	NLRP12	–	–	–
	NLRC4	Truncated	–	–
PYHIN	AIM2	Absent	Absent	Absent
	IFI16	Truncated	–	–
GSDM	GSDMA	–	–	–
	GSDMB	Mutation	Mutation	Mutation
	GSDMC	–	–	–
	GSDMD	–	–	–
	GSDME	–	Mutation	Mutation

“-” means no deficiency.

Inflammasomes produce inflammatory cytokines and induce pyroptosis in response to intracellular danger-associated signals. NLRP3 inflammasome plays an important role for the pathogenesis of severe COVID-19 (11). Thus, NLRP3 inflammasome and pyroptosis pathways are considered as attractive targets for therapy of COVID-19 with severe symptoms (39). Mouse hepatitis virus (MHV) could activate the NLRP3 inflammasome and inflammatory cell death. However, deleting NLRP3 or GSDMD led to an initial reduction in cell death followed by a robust increase of inflammatory cell death after MHV infection (40). Thus, balance of cell death and inflammatory immune responses is critical to promote protection against coronavirus infection. SARS-CoVs protein E, open reading frame 3a (ORF3a) and ORF8a are able to activate NLRP3 inflammasomes (41–44). Although NLRP3 in canines and felines are intact, GSDM family proteins, the critical components of pyroptosis pathways induced by inflammasome activation, are deficient. GSDMB is cleaved by CASP1 at D236 (32). Dogs, cats and tigers lose this key residue D. The critical amino acid for granzyme A cleavage in GSDMB is K244 (33). Dogs and tigers lose this key residue. Thus, the activation sites in GSDMB were deficient in felines and canines, indicating the deficiency of GSDMB-dependent pyroptosis pathway. The GSDME activation site in felines was also abolished. Thus, some pyroptosis pathways in canines and felines are deficient, which might partially account for the suppression of inflammation induced by inflammasome activation in canines and felines.

Viruses potently drive the evolutionary adaptations in their hosts. Bats and pangolin have dampened antiviral responses, indicating that they have adapted to the evolutionary pressure exerted by viruses through decreasing inflammatory responses. The results of the present study suggested that canines and felines evolutionarily down-regulated the inflammasome and pyroptosis pathways. We speculated that excessive exposure to cytosolic DNA in canines and felines during viral infection would pose a natural selection pressure to suppress the activation of inflammasome and pyroptosis pathways.

Although few reports of natural SARS-CoV-2 infection in canines/felines are documented, the number of naturally infected canines/felines is very low as compared to humans. We previously showed dog has a soluble isoform of ACE2, which could block the interaction between full length ACE2 and S (45). That’s one important reason for the low number of naturally infected dogs. Another reason is lack of large scale screen for SARS-CoV-2 infection in canines and felines. At the same time, a large number of asymptomatic SARS-CoV-2 infected humans are detected, and most young people have no or mild symptoms. We propose that deficiency in inflammasome and pyroptosis pathways is only responsible for reduced clinical response in canines and felines. Host immune genes diversity might be the possible reason for asymptomatic humans. For instance, inflammasome response is increased as people get old, which is correlated to the more severe symptoms in old people.

Although the presence or absence of inflammasome and pyroptosis genes is important for clinical symptoms of COVID-19, we cannot fully predict the consequences *in vivo*. Future experimental studies in animals or in cells isolated from animals will be necessary to test our mechanistic hypotheses.

In summary, canines/felines and humans differ in the key components of inflammasome and pyroptosis pathways, which are associated with the responses to SARS-CoV-2 infection. Some components are missing or truncated, others are not activated. The deficiency of inflammasome and pyroptosis pathways in those mammals may decrease excessive inflammation and hence increases disease tolerance. Our study not only extends the understanding of the evolution of inflammasome and pyroptosis, but also has implications for interpreting a symptom or mild symptom in cats, dogs and tigers infected with SARS-CoV-2.

## Data Availability Statement

The data that support the findings of this study are available from the corresponding author upon reasonable request.

## Author Contributions

LZ designed the study. HC performed bioinformatics analyses. HC and LZ wrote the manuscript. All authors contributed to the article and approved the submitted version.

## Funding

This work was supported by grants from National Key Plan for Research and Development of China [2016YFD0500300], National Natural Science Foundation of China [81871663 and 82072270], and Academic promotion programme of Shandong First Medical University [2019LJ001].

## Conflict of Interest

The authors declare that the research was conducted in the absence of any commercial or financial relationships that could be construed as a potential conflict of interest.
