# Supplementary material for: Key Components of Inflammasome and Pyroptosis Pathways Are Deficient in Canines and Felines, Possibly Affecting Their Response to SARS-CoV-2 Infection
Source: Front Immunol. 2021 Jan 28;11:592622. doi: 10.3389/fimmu.2020.592622 (PMC7876337; doi:10.3389/fimmu.2020.592622)
Supplement: Supplementary file 1 [file DataSheet_1.docx]

**Key components of inflammasome and pyroptosis are deficient in canines and felines, implying the mild symptoms of SARS-CoV-2 infection**

**Haoran Cui, Leiliang Zhang**

**Supplemental Figures legends**

**Figure S1.** Sequence alignment of dog IFI16 isoforms and human IFI16.

**Figure S2.** Protein sequence alignment of NLRP3.

**Figure S3.** mRNA sequence alignment of NLRP3.

**Figure S4.** Protein sequence alignment of NLRP6.

**Figure S5.** mRNA sequence alignment of NLRP6.

**Figure S6.** Protein sequence alignment of NLRP9.

**Figure S7.** mRNA sequence alignment of NLRP9.

**Figure S8.** Protein sequence alignment of NLRP12.

**Figure S9.** mRNA sequence alignment of NLRP12.

**Figure S10.** Protein sequence alignment of NLRP1.

**Figure S11.** mRNA sequence alignment of NLRP1.

**Figure S12.** Genome data viewer of tiger genome in the corresponding site of cat NLRP1.

**Figure S13.** Genome data viewer of tiger genome in the corresponding site of human NLRP1.

**Figure S14.** Protein sequence alignment of NLRC4.

**Figure S15.** mRNA sequence alignment of NLRC4.

**Figure S16.** Protein sequence alignment of ASC.

**Figure S17.** mRNA sequence alignment of ASC.

**Figure S18.** Protein sequence alignment of caspase-1.

**Figure S19.** mRNA sequence alignment of caspase-1.

**Figure S20.** Protein sequence alignment of GSDMA.

**Figure S21.** mRNA sequence alignment of GSDMA.

**Figure S22.** Protein sequence alignment ofGSDMC.

**Figure S23.** mRNA sequence alignment of GSDMC.

**Figure S24.** Protein sequence alignment of GSDMD.

**Figure S25.** mRNA sequence alignment of GSDMD.

**Figure S26.** Protein sequence alignment of GSDMB.

**Figure S1.** Sequence alignment of dog IFI16 isoforms and human IFI16.

**
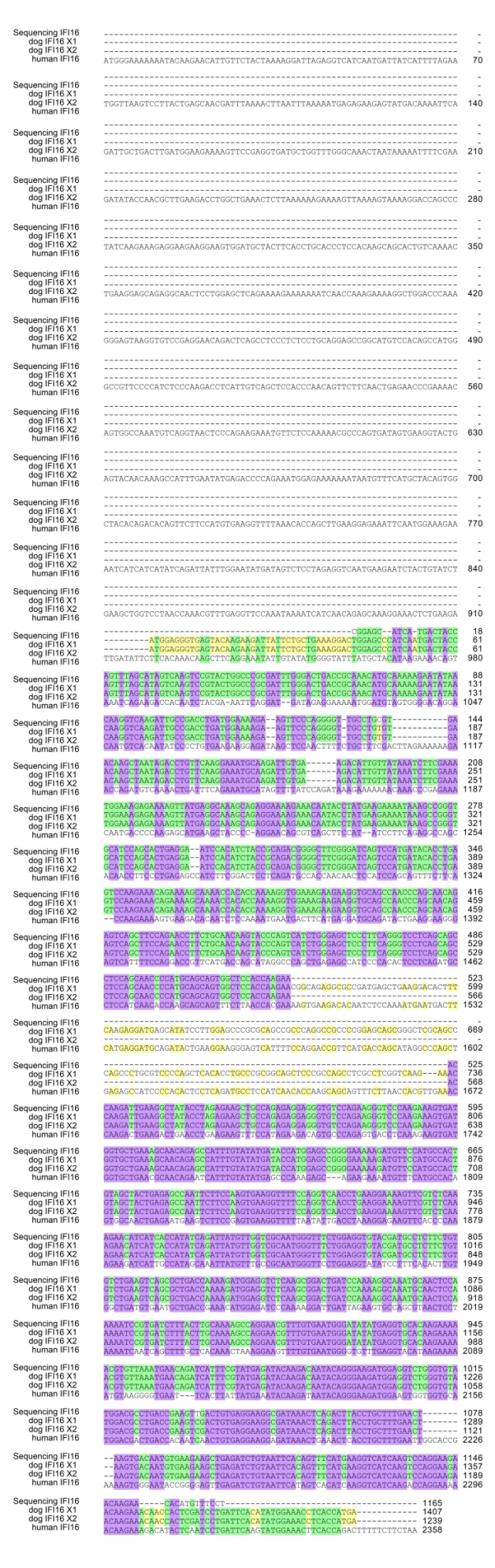
**

**Figure S2.** Protein sequence alignment of NLRP3.

**
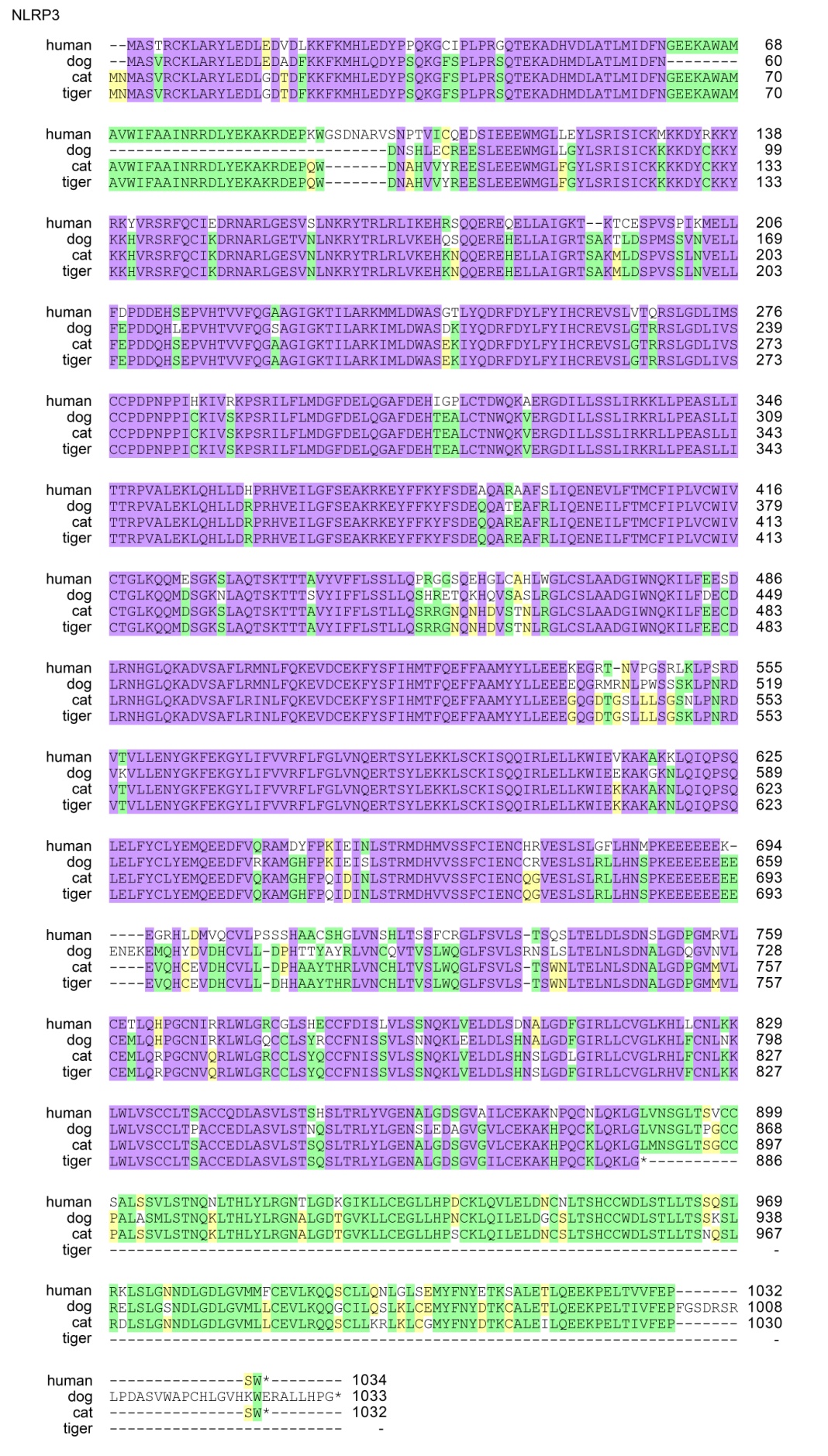
**

**Figure S3.** mRNA sequence alignment of NLRP3.

**
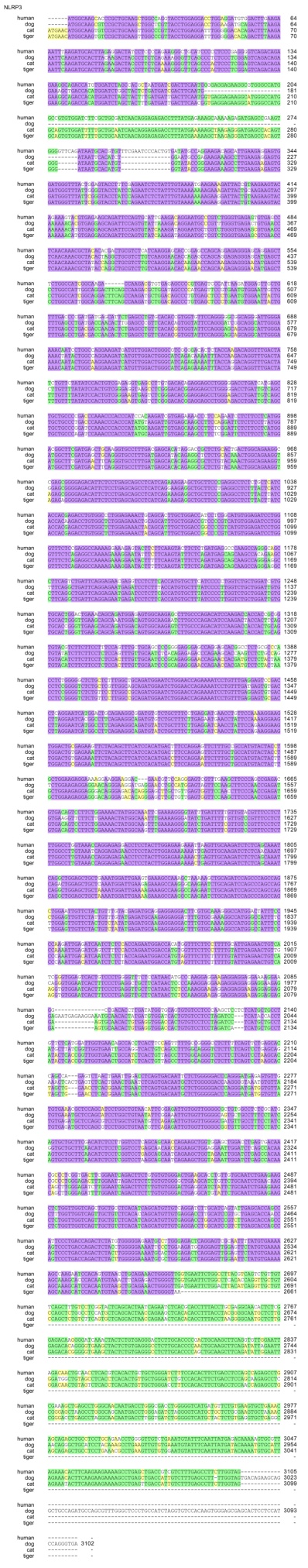
**

**Figure S4.** Protein sequence alignment of NLRP6.

**
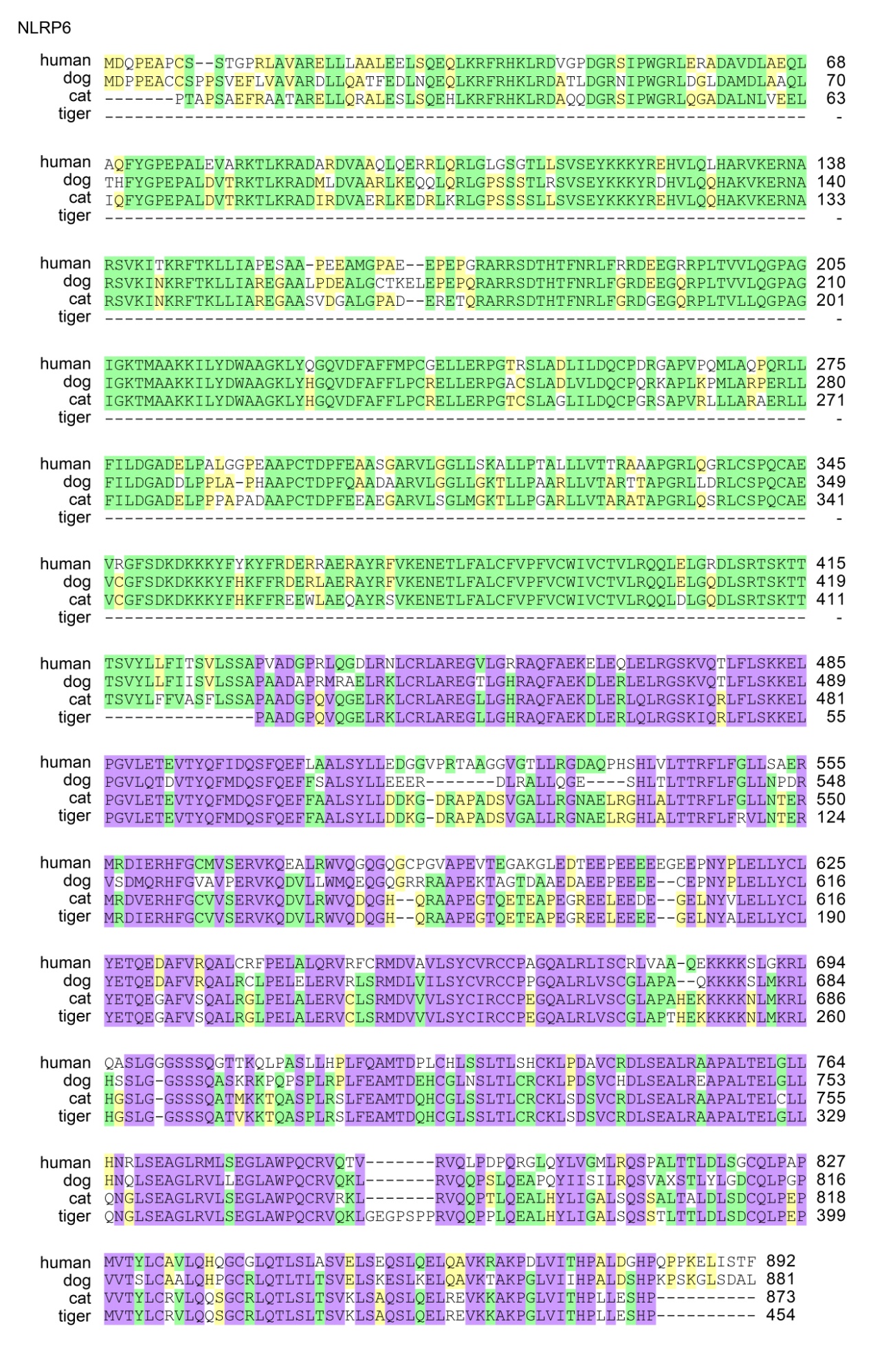
**

**Figure S5.** mRNA sequence alignment of NLRP6.

**
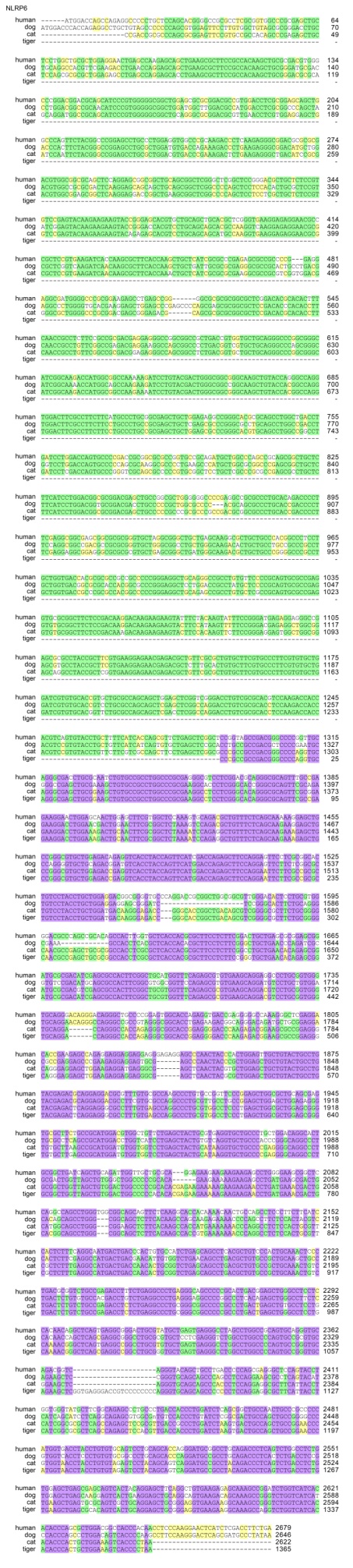
**

**Figure S6.** Protein sequence alignment of NLRP9.

**
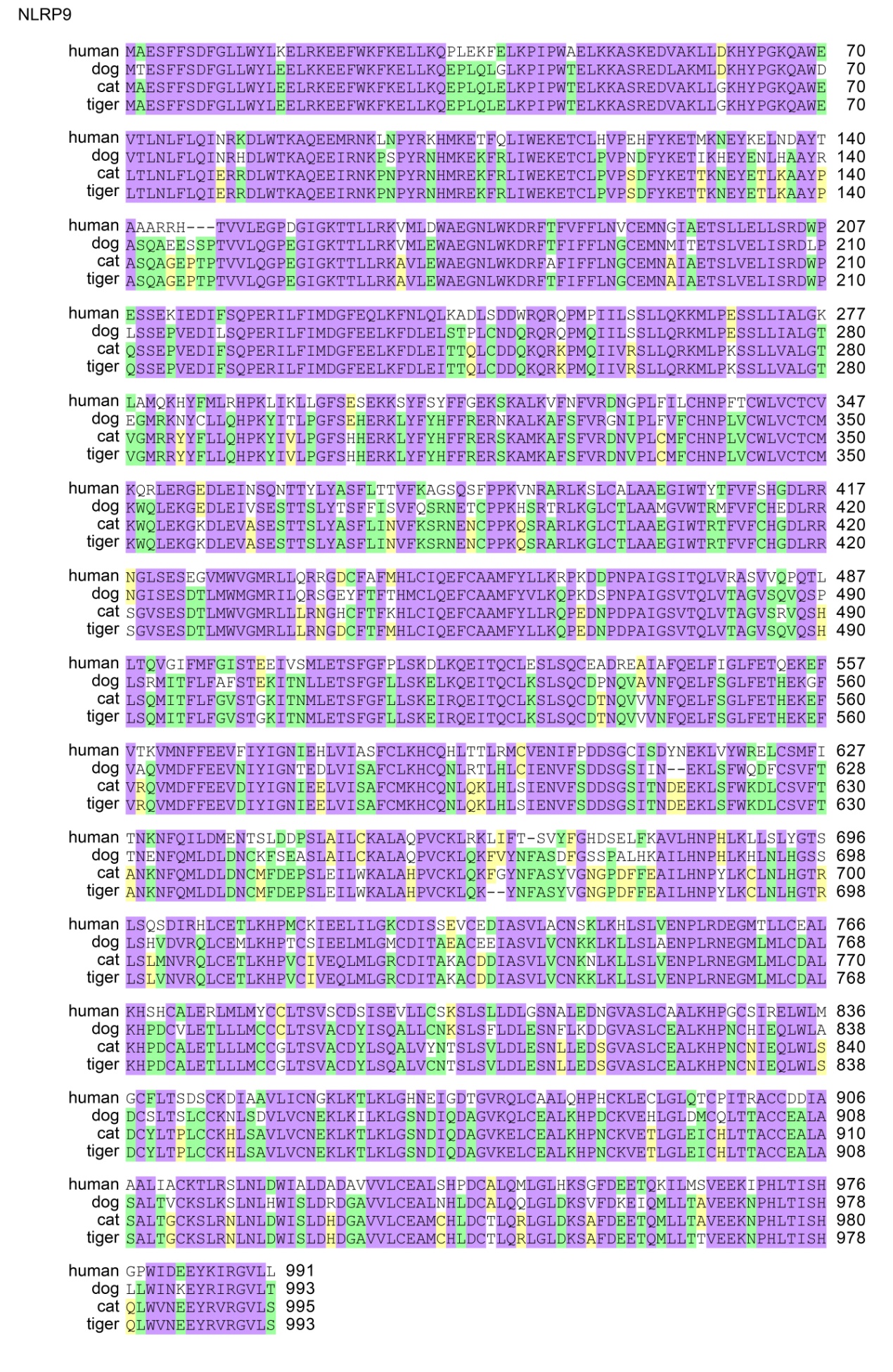
**

**Figure S7.** mRNA sequence alignment of NLRP9.

**
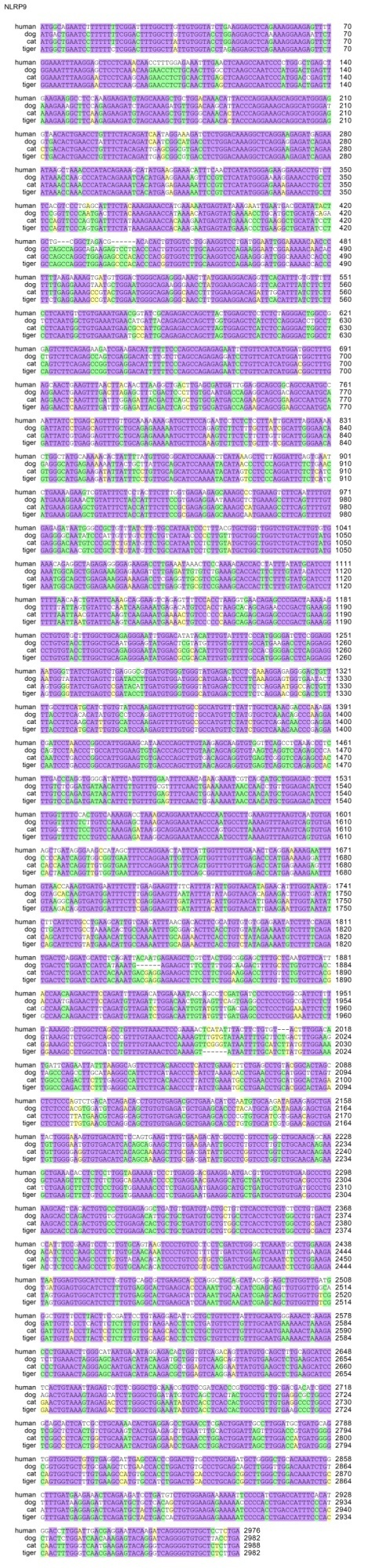
**

**Figure S8.** Protein sequence alignment of NLRP12.

**
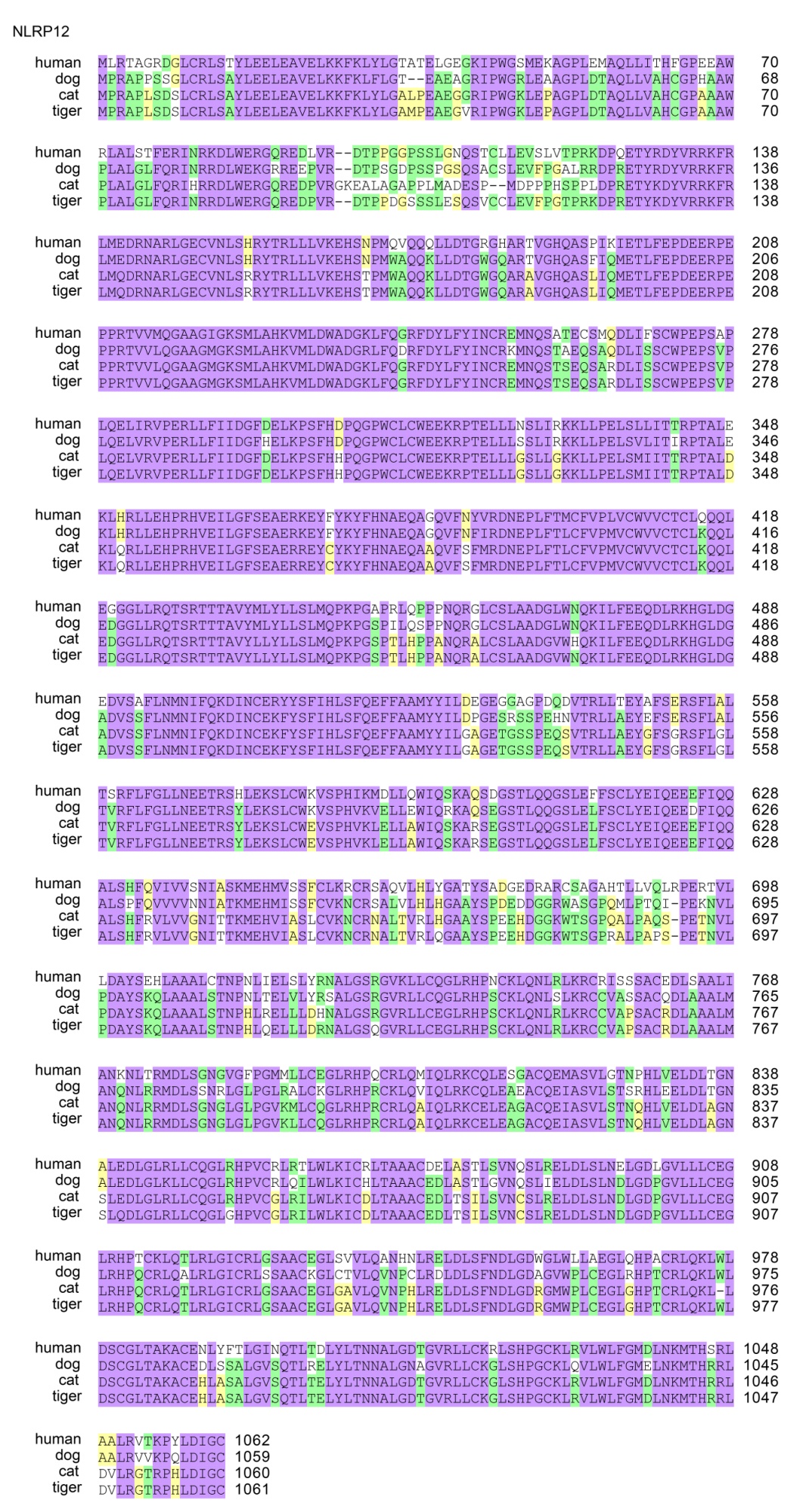
**

**Figure S9.** mRNA sequence alignment of NLRP12.

**
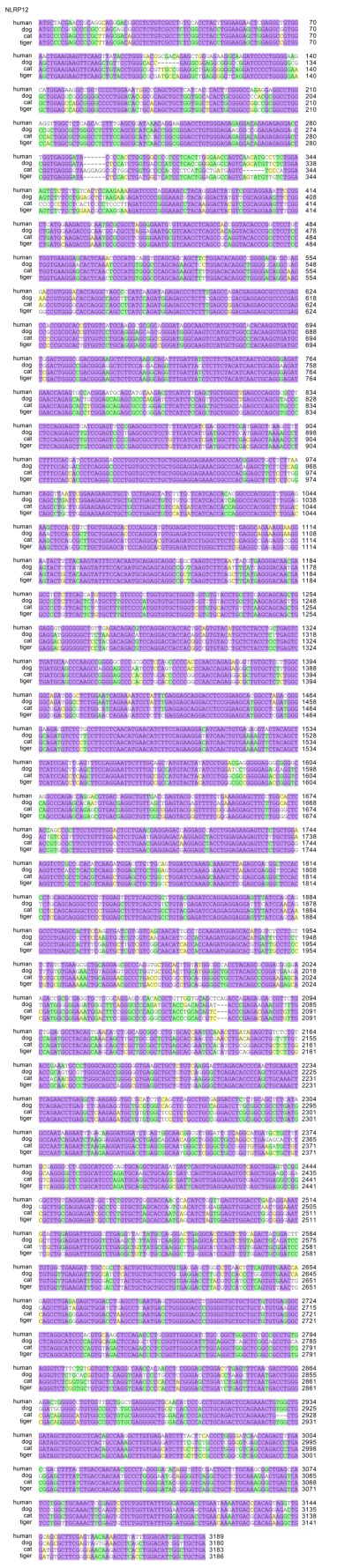
**

**Figure S10.** Protein sequence alignment of NLRP1.


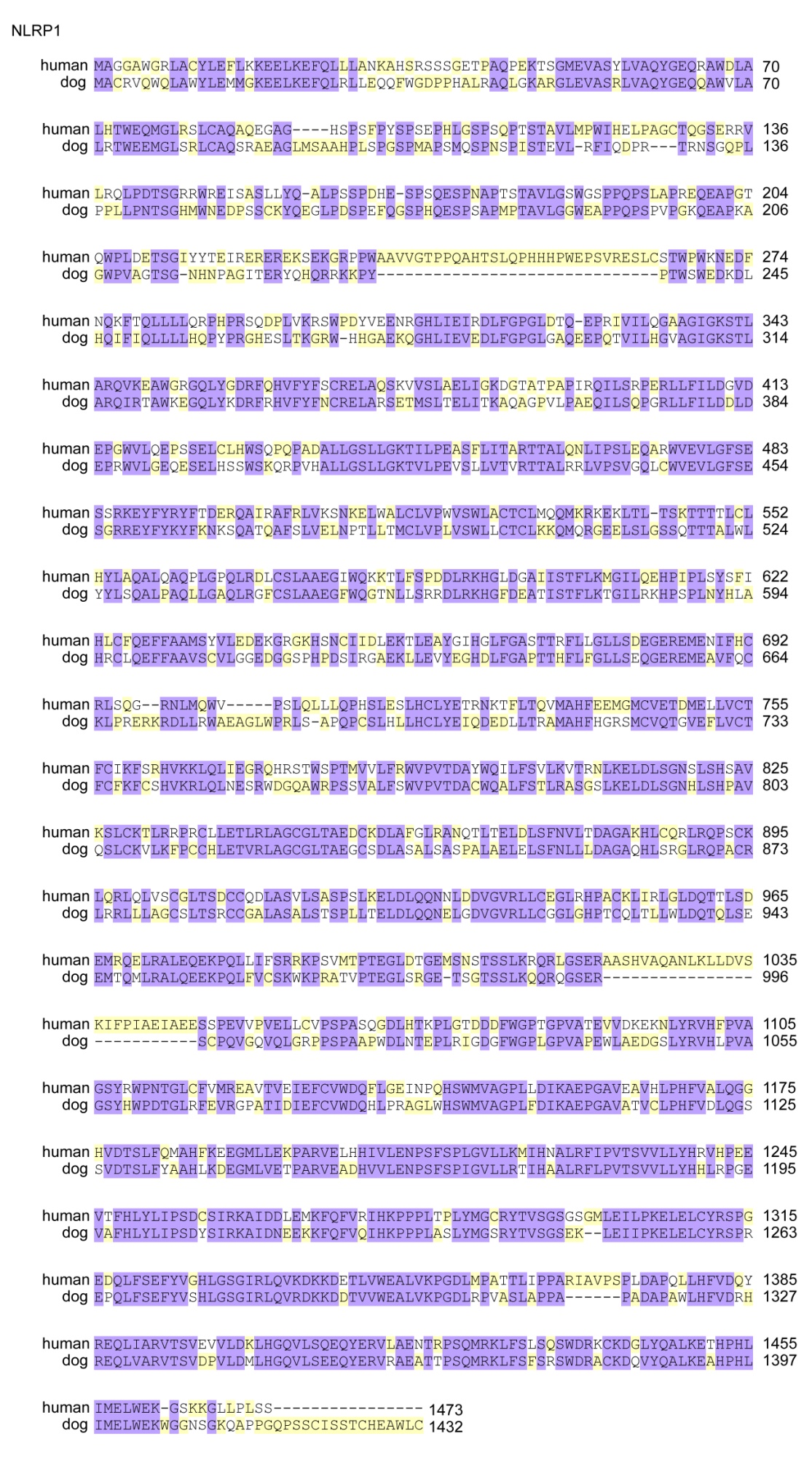


**Figure S11.** mRNA sequence alignment of NLRP1.

**
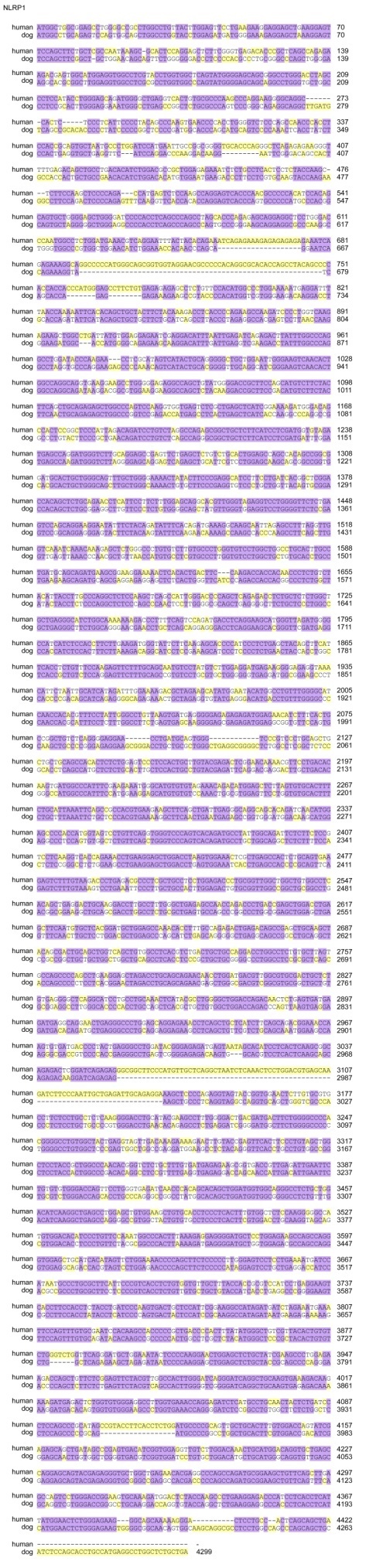
**

**Figure S12.** Genome data viewer of tiger genome in the corresponding site of cat NLRP1.

**
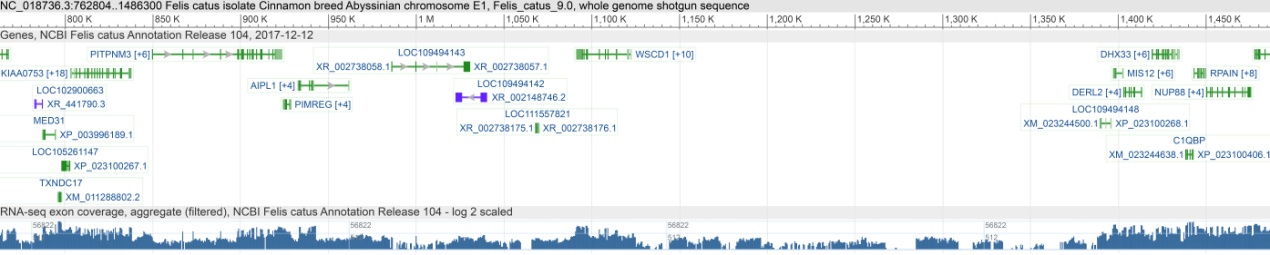
**

**Figure S13.** Genome data viewer of tiger genome in the corresponding site of human NLRP1.

**
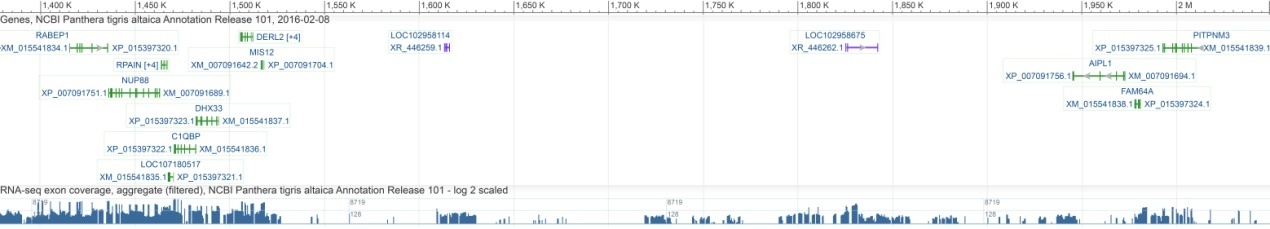
**

**Figure S14.** Protein sequence alignment of NLRC4.

**
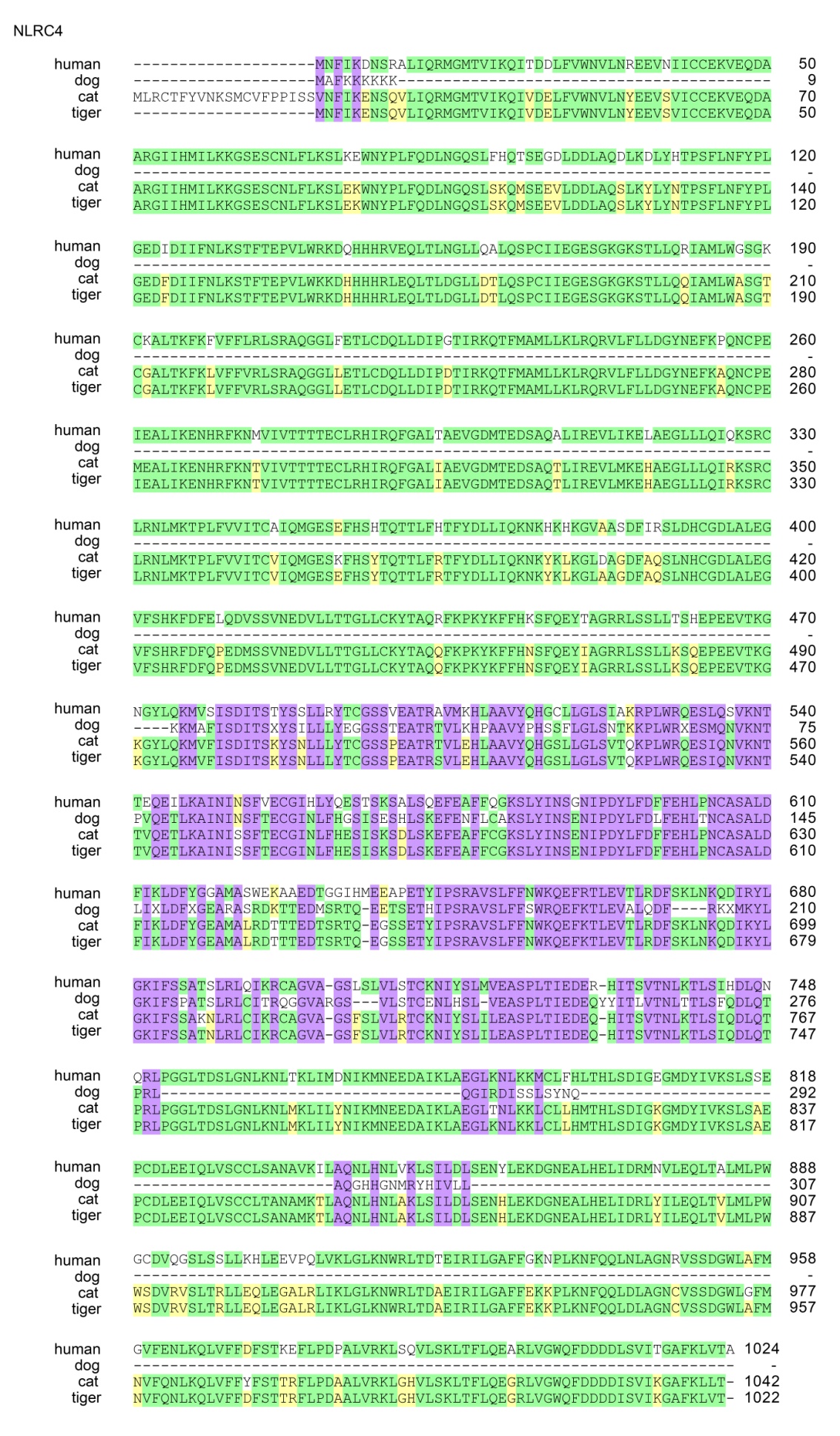
**

**Figure S15.** mRNA sequence alignment of NLRC4.


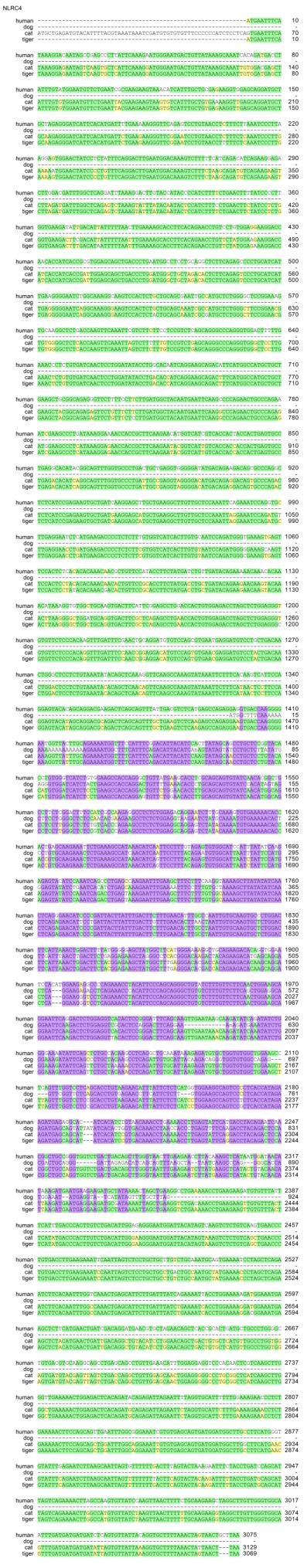


**Figure S16.** Protein sequence alignment of ASC.

**
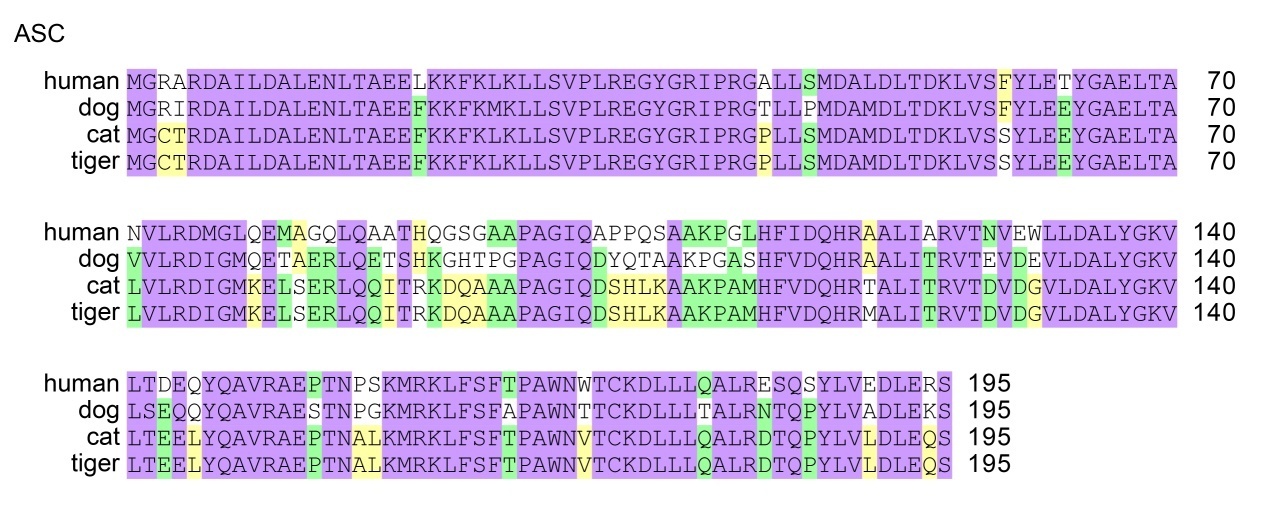
**

**Figure S17.** mRNA sequence alignment of ASC.

**
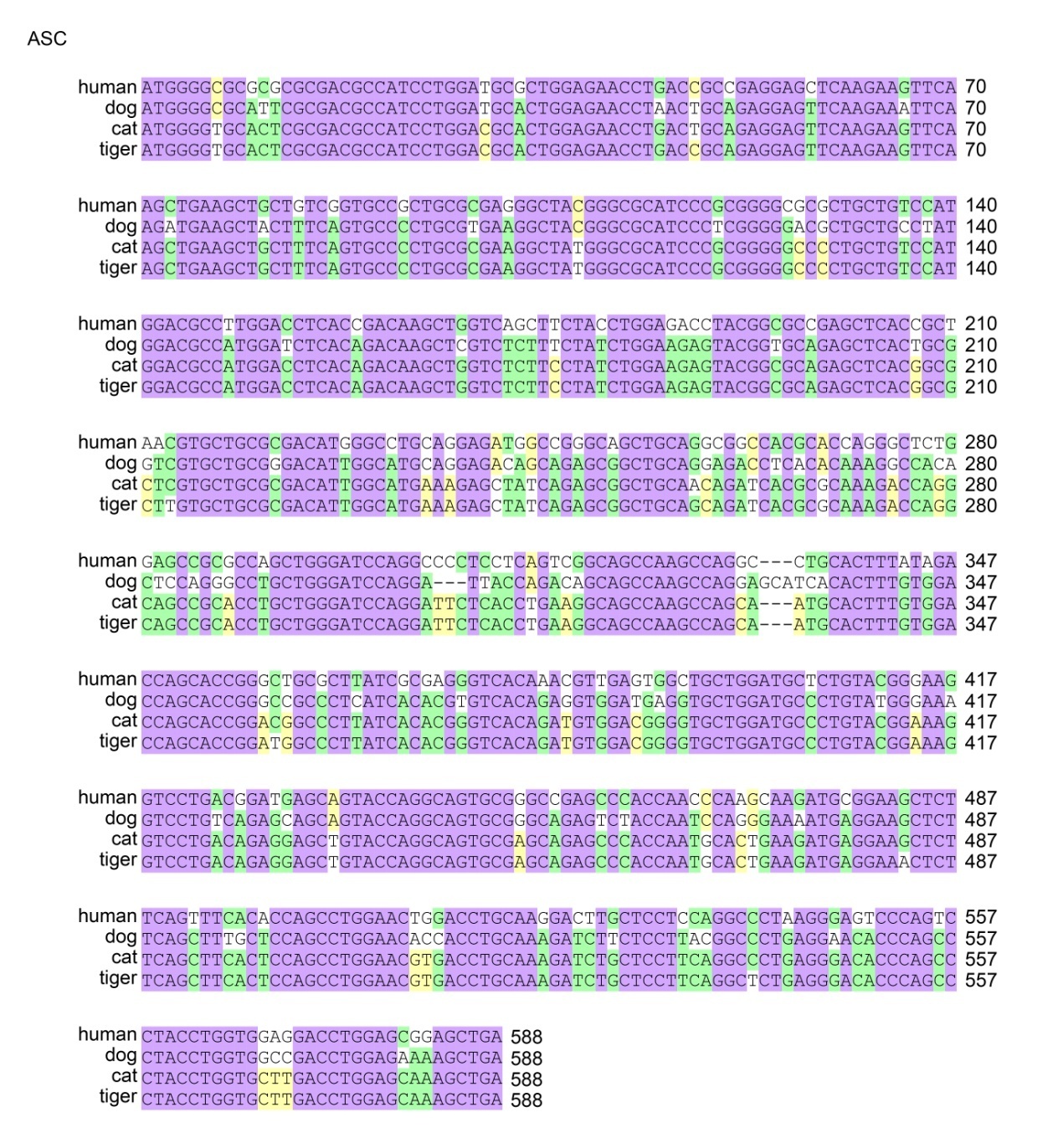
**

**Figure S18.** Protein sequence alignment of caspase-1.

**
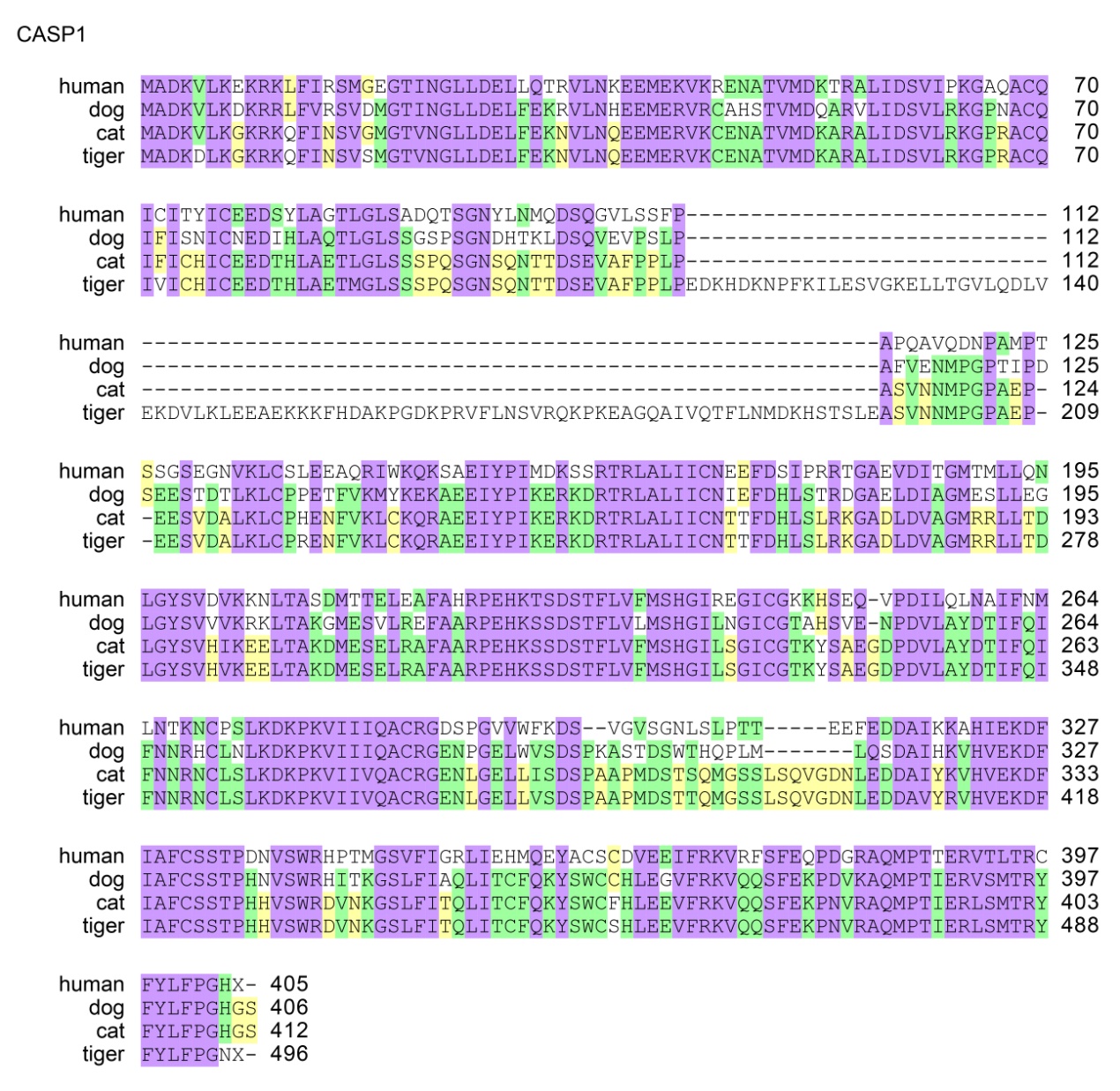
**

**Figure S19.** mRNA sequence alignment of caspase-1.

**
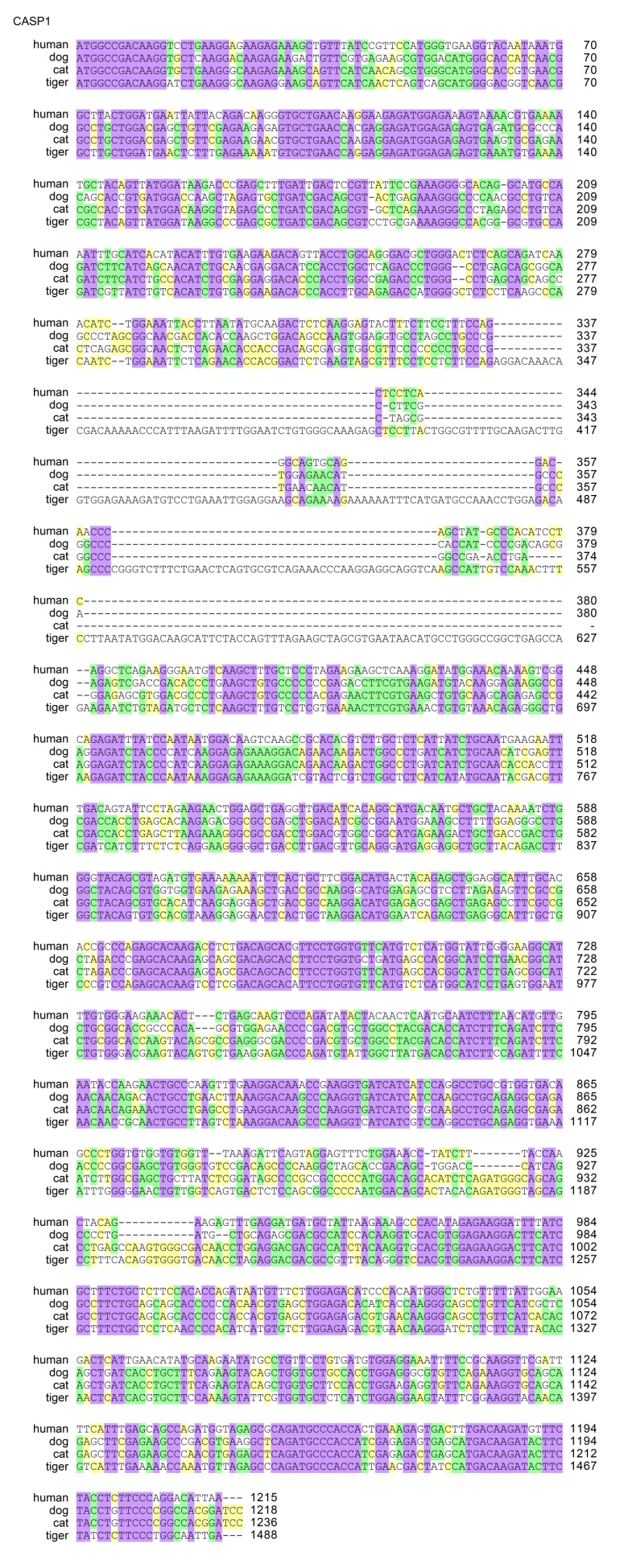
**

**Figure S20.** Protein sequence alignment of GSDMA.

**
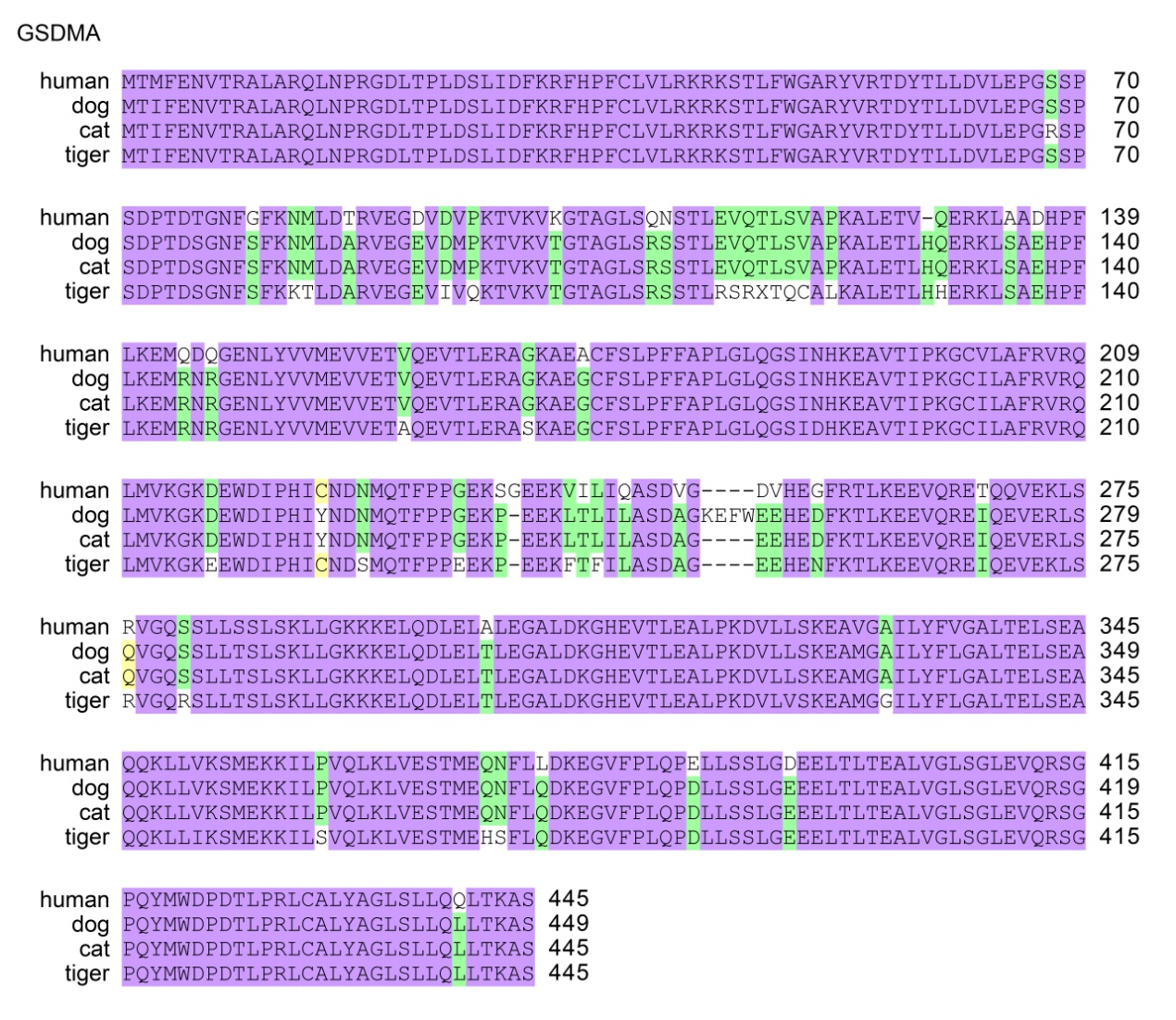
**

**Figure S21.** mRNA sequence alignment of GSDMA.

**
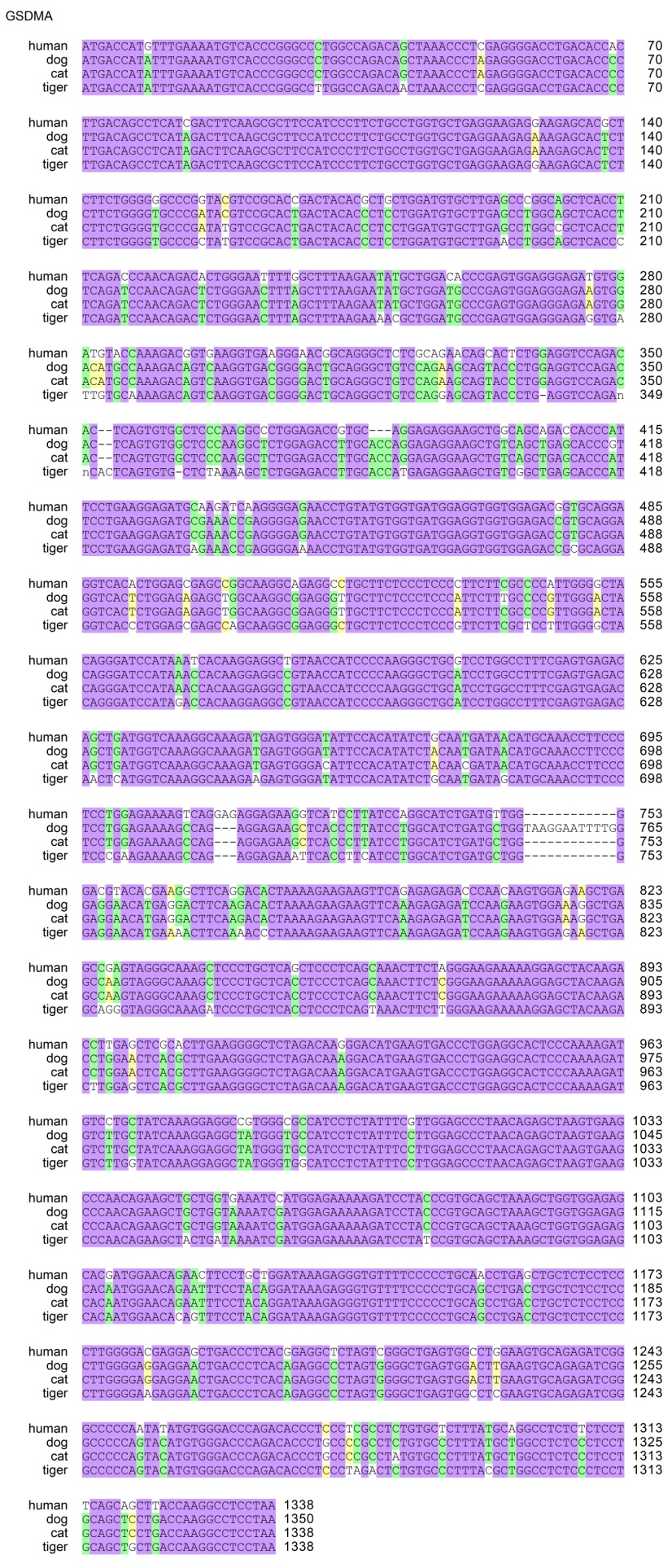
**

**Figure S22.** Protein sequence alignment ofGSDMC.

**
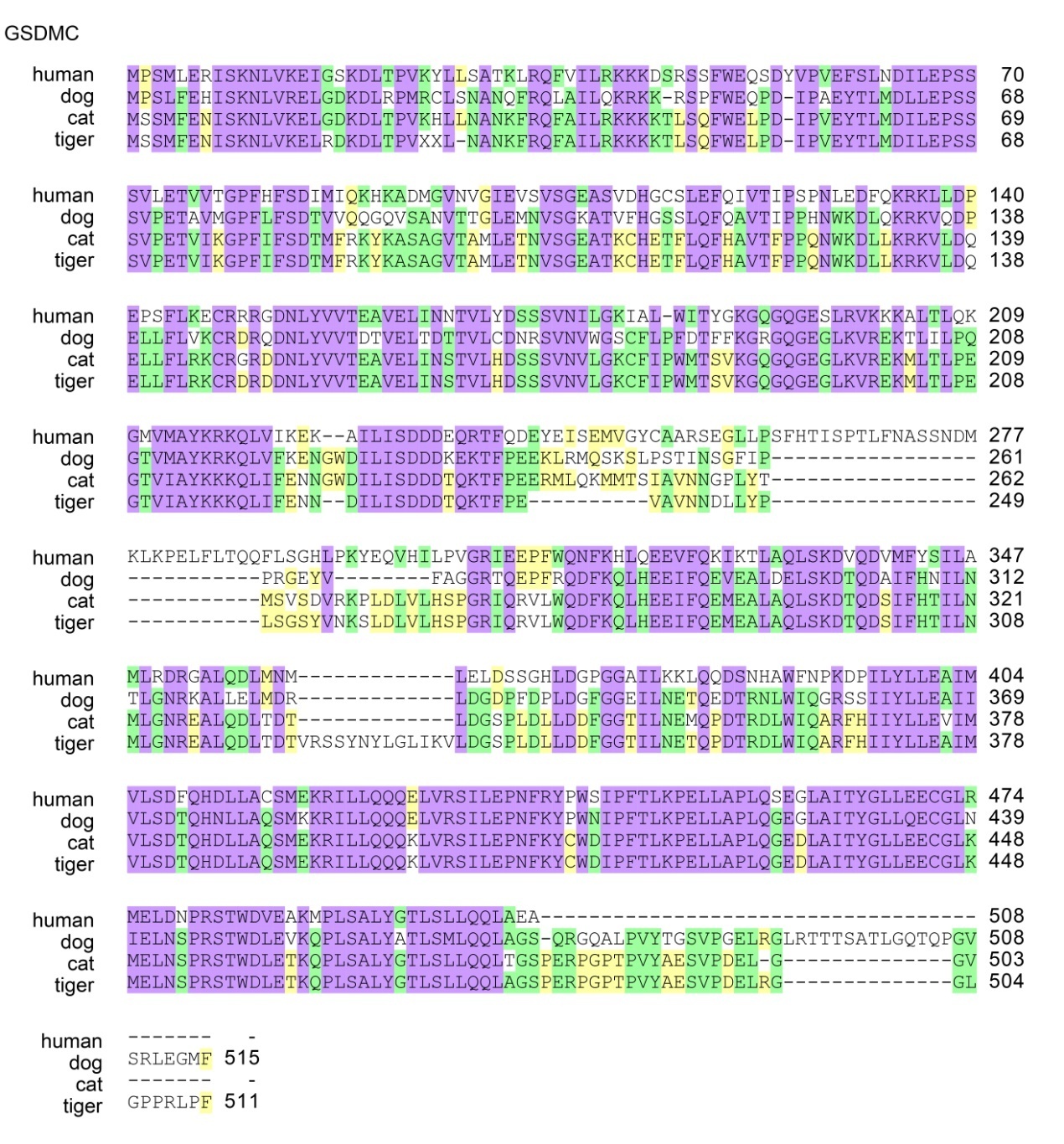
**

**Figure S23.** mRNA sequence alignment of GSDMC.

**
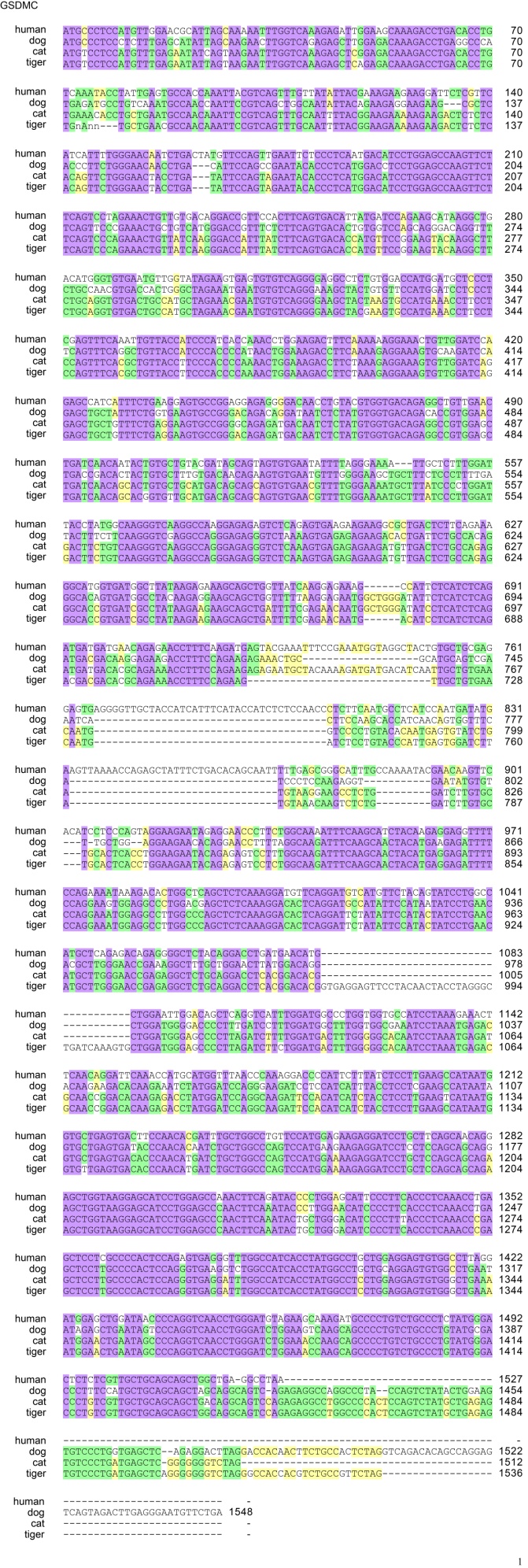
**

**Figure S24.** Protein sequence alignment of GSDMD.

**
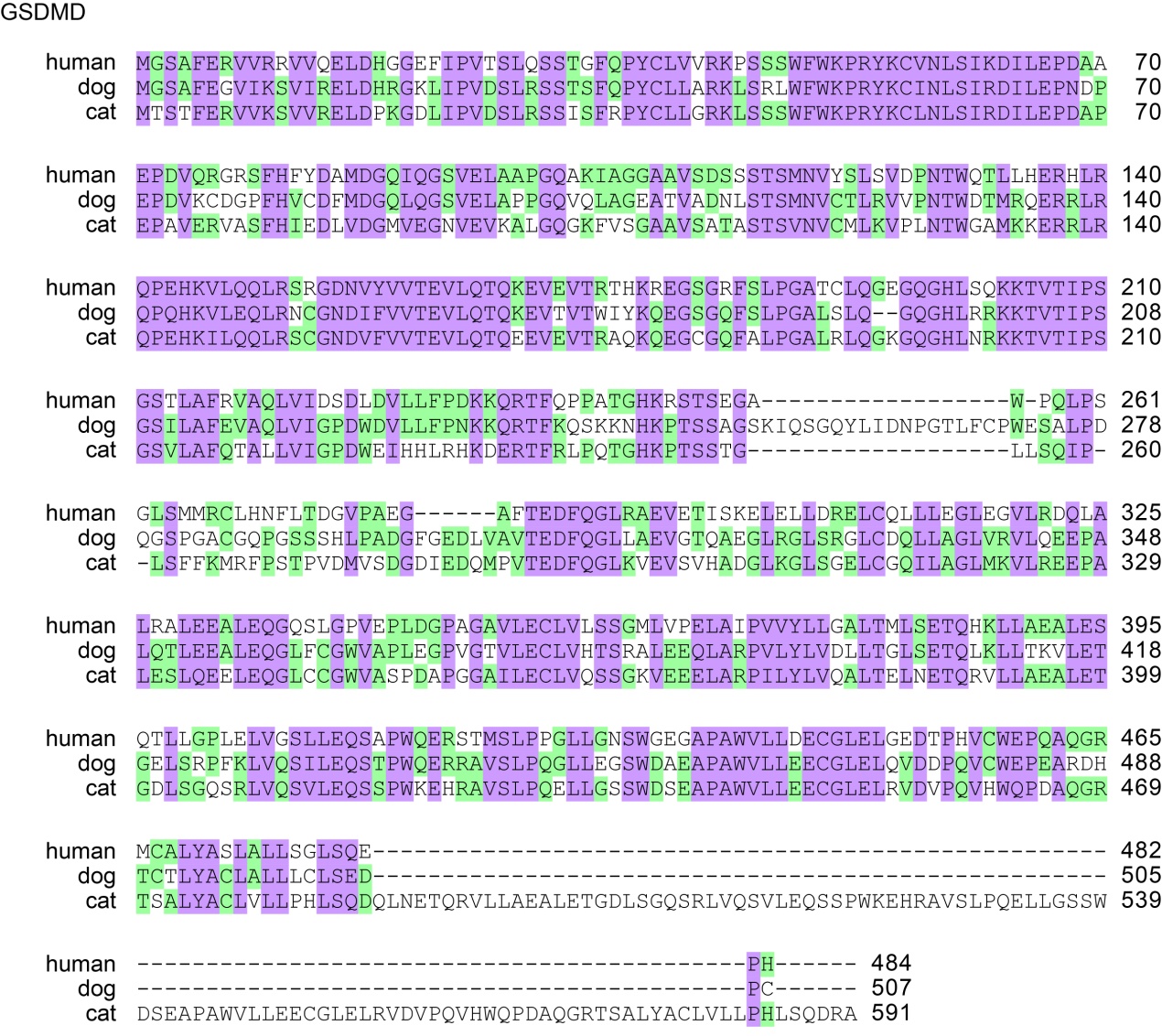
**

**Figure S25.** mRNA sequence alignment of GSDMD.

**
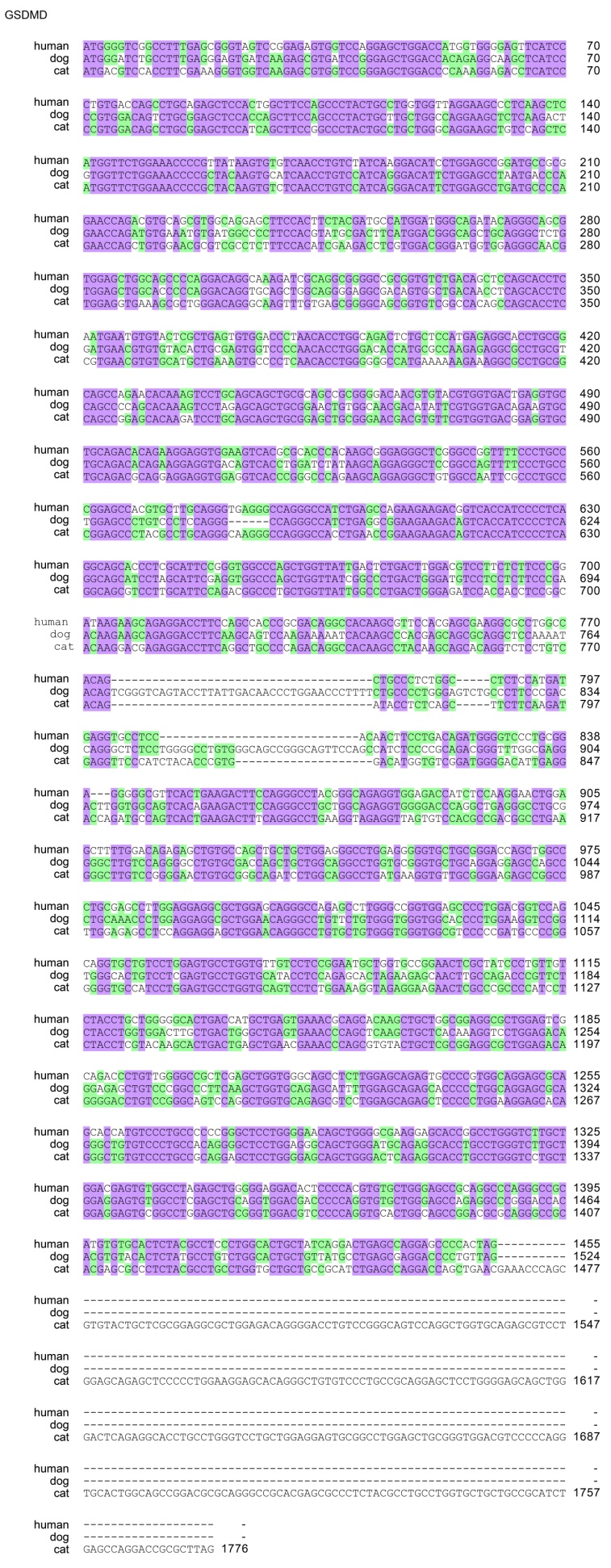
**

**Figure S26.** Protein sequence alignment of GSDMB.

**
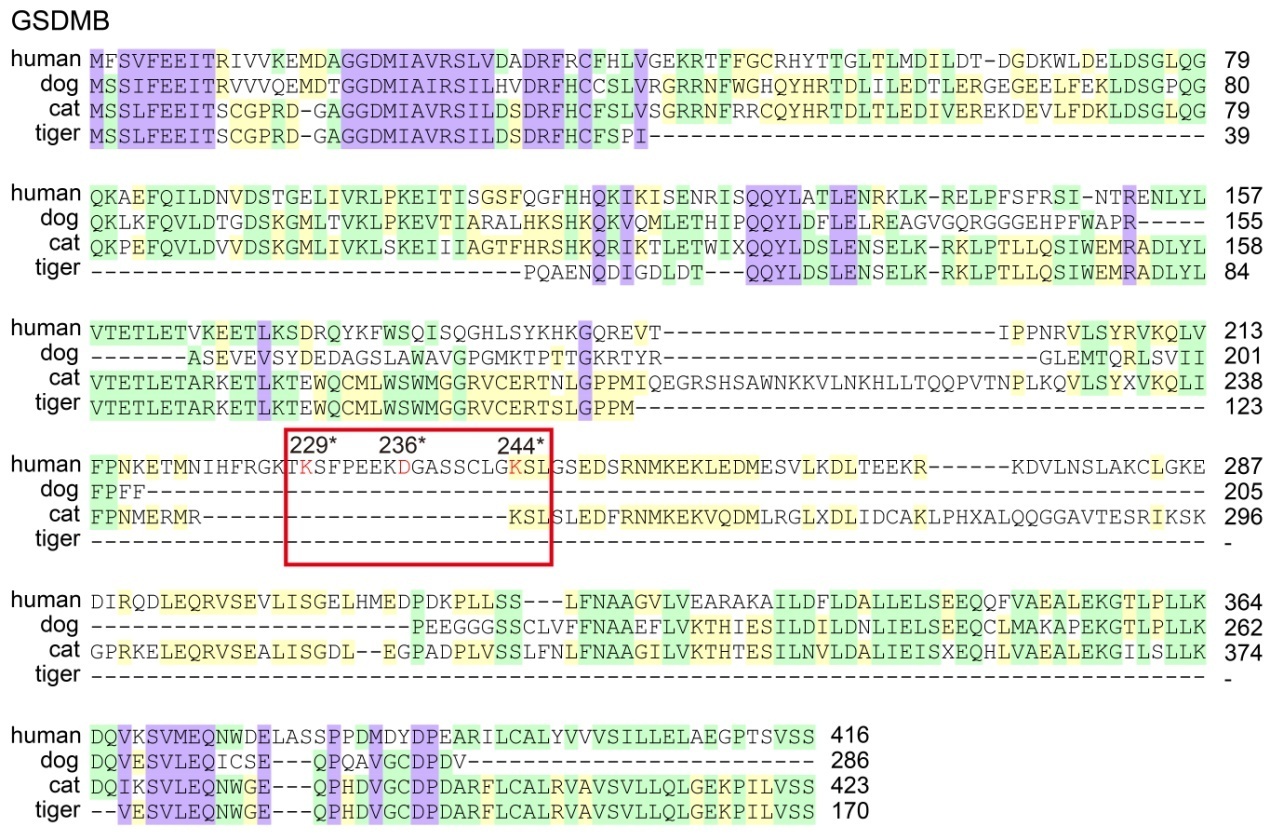
**
